# Empowering differential networks using Bayesian analysis

**DOI:** 10.1371/journal.pone.0261193

**Published:** 2022-01-25

**Authors:** Jarod Smith, Mohammad Arashi, Andriëtte Bekker

**Affiliations:** 1 Department of Statistics, University of Pretoria, Pretoria, South Africa; 2 Department of Statistics, Faculty of Mathematical Sciences, Ferdowsi University of Mashhad, Mashhad, Iran; Central European University, HUNGARY

## Abstract

Differential networks (DN) are important tools for modeling the changes in conditional dependencies between multiple samples. A Bayesian approach for estimating DNs, from the classical viewpoint, is introduced with a computationally efficient threshold selection for graphical model determination. The algorithm separately estimates the precision matrices of the DN using the Bayesian adaptive graphical lasso procedure. Synthetic experiments illustrate that the Bayesian DN performs exceptionally well in numerical accuracy and graphical structure determination in comparison to state of the art methods. The proposed method is applied to South African COVID-19 data to investigate the change in DN structure between various phases of the pandemic.

## Introduction

Probabilistic networks are becoming ever-present in a multitude of scientific disciplines. These networks aim to illustrate the relationships, if any, between the components of complex systems [1]. If the data is assumed to be Gaussian distributed with mean ***μ*** and covariance matrix **Σ**; the precision matrix **Θ** ≔ {*θ*_*ij*_}, defined as the inverse of the covariance matrix **Θ** ≡ **Σ**^−1^, directly determines the conditional dependence relations and structure of the Gaussian undirected graphical model [2].

Differential network (DN) analysis is a statistical methodology that involves functions of at least two graphical models. Let G=(V,E) define a graphical model with nodes V={1,2,...,p} and a set of edges E⊆V×V. The graph visually depicts the conditional dependence structure between the nodes of the system. The adjacency matrix associated to a graphical model G is the binary encoded *p* × *p* precision matrix where the entries of the matrix are equal to 1 if the corresponding precision matrix entry is nonzero and zero otherwise. Nonzero adjacency matrix entries indicate an edge between corresponding nodes of G. For this work, the focus will be on the difference of two Gaussian graphical models (GGM), $G_{1}$ and $G_{2}$ that share the same set of nodes V. In particular, the edge sets given here are equivalent to the adjacency matrices obtained from the GGM estimation. More specifically, assume that the observations, $x_{1},x_{2},\ldots ,x_{n_{1}}$ and $y_{1},y_{2},\ldots ,y_{n_{2}}$ are generated from a *p* variate Gaussian distribution, *N*_*p*_(***μ***_1_, **Σ**_1_) and *N*_*p*_(***μ***_2_, **Σ**_2_), respectively, where *n*_1_ and *n*_2_ indicate the respective sample sizes that need not be equal. The interest here is estimating the DN ($\Delta =\Sigma_{2}^{-1}-\Sigma_{1}^{-1}$), that is the difference between two precision matrices. Numerous measures exist for comparing and evaluating the differences between graphical structures [1]. DN analysis is becoming increasingly popular and important, for example in biological systems where protein interaction networks can be utilised as informative biosignatures for prevalent diseases [3, 4]. The fundamental idea here is that, if two molecules interact with one another then a statistical dependency between them should be observed. Additionally, another application of DNs is multivariate statistical quadratic discriminant analysis [5, 6], under the Gaussian distribution assumption.

A key component of DN analysis is the estimation of covariance and precision matrix components. Numerous statistical matrix estimation, as well as graphical model determination methods exist within literature. In particular, from a frequentist approach [7], introduce a computationally efficient neighborhood selection procedure. The lasso is used for covariance estimation which enjoys consistency for sparse high-dimensional graphs. The approach is quite effective, in that the sparse precision matrix is estimated by fitting the lasso to each variable using the remaining as predictors. Finally, the estimated precision matrix entry (*θ*_*ij*_) is non-zero if the estimated coefficient of *i* on *j* or vice versa is non-zero. Importantly, their algorithm can consistently estimate the set of non-zero entries in **Θ**, [8]. For a penalised likelihood methodology for sparse precision matrix estimation see [9, 10]. More so [11], estimate the undirected graphical model using both a block coordinate descent algorithm, as well as Nesterov’s first order method [12]. Additionally [13], propose a *ℓ*_1_ constraint estimation technique for both sparse and non-sparse high dimensional matrices with applicability on a wide range of sparsity patterns and class of matrices; precision estimation in GGMs for example. For a joint graphical model estimation approach see [14, 15].

Fully Bayesian treatments of GGM estimation are, also, well rooted in literature. In particular [16], introduce the Bayesian adaptive graphical lasso (BAGLASSO) which utilises a generalised Pareto distribution in the hierarchical formulation of the Bayesian graphical lasso. [17] provide a method for graphical model determination by invoking positive prior mass on the event that there is no conditional dependencies between variables. In terms of joint graphical model inference from a Bayesian perspective see [18]. Lastly [19], propose using Kullback-Leibler divergence and cross-validation for graphical model structure estimation.

### Background

Recently, a plethora of statistical techniques have emerged for estimating DNs. These techniques can largely be classified into two main categories. The first estimating the individual precision matrices, **Θ**_1_ and **Θ**_2_ separately; where the estimated DN is the difference between the estimated precision matrices. For example, the methods and references for GGM estimation outlined in the introduction can be used to directly estimate **Δ**. The second methodology estimates both the precision matrices simultaneously. The approach here, typically penalises a joint loss function for both precision matrices. [20] provide a methodology for inference and estimation of functions of GGMs. In particular, the Intertwined Graphical Lasso (IGL) approach biases the estimation of the precision matrices towards a common value. More so, their Graphical Cooperative Lasso (GCL) utilises a group-penalty for solutions that favour a common sparsity pattern. [14, 21] estimate separate graphical models using a joint penalised loss function. [22] propose a method for estimating **Δ** directly which relaxes the need for both individual precision matrices to be sparse nor be estimated directly. Similarly [6, 23], utilise an alternating direction method of multipliers (ADMM) algorithm for estimating **Δ** from their joint *ℓ*_1_ penalised convex loss function. More recently [24], introduce a computationally efficient iterative shrinkage-thresholding algorithm for minimising the *ℓ*_1_ loss function defined in [6], namely

$$\begin{array}{c} L_{1}\left(\Delta \right)=\frac{1}{2}\text{trace}\left(\Delta^{⊤}S_{1}\Delta S_{2}\right)-\text{trace}\left(\Delta \left(S_{1}-S_{2}\right)\right), \end{array}$$ (1)

is convex and **S**_1_ and **S**_2_ are the sample covariance matrices. The DN estimate is obtained by minimising the penalised loss Eq (1). An analogous symmetric convex loss function and estimator is proposed by [23].

The shrinkage-thresholding algorithm proposed by [24], based on the fast-iterative shrinkage-thresholding algorithm in [25], aims to minimise Eq (1). The objective function is given by

$$\begin{array}{c} \underset{\Delta \in R^{p\times p}}{\text{arg}\;\text{min}}L_{1}\left(\Delta \right)+\rho {\left\|\Delta \right\|}_{1}, \end{array}$$

where ${\left\|\Delta \right\|}_{1}=\sum_{i<j}\sum_{i=1}^{p}\left|{\hat{\Theta }}_{2:ij}-{\hat{\Theta }}_{1:ij}\right|$. The lasso tuning parameter, *ρ*, controls the strength of the penalty term and resultantly the amount of shrinkage (precision matrix entries shrunk towards zero) too. The optimisation objective converges to the solution sequentially using a quadratic approximation and a gradient descent algorithm. The efficiency of the procedure is attested to this approach, resulting in superior computational complexity in contrast to the ADMM approaches by [6, 23]. To conclude this section it is worth noting that the iterative shrinkage-thresholding method will be used for experimental comparison later.

The main contributions of this study are as follows.

1. A framework for Bayesian DN estimation is developed. That is, the DN is estimated by separately estimating each Gausian graphical model, referred to as the components.
2. The graphical lasso is applied as the thresholding method in the Bayesian precision matrix estimation in order to efficiently capture sparse patterns in the DN, hence developing the BAGLASSO. A threshold selection strategy, based on a conjugate Wishart prior, that accommodates both dense and sparse graphical structures determination is explored. The aforementioned strategy, applied to each component of the DN, ensures an accurately sparse DN estimate.
3. The proposed Bayesian DN efficiently improves the existing classical DN estimation for a number of known network structures.
4. An R package for the BAGLASSO block Gibbs sampler has been developed for the interested practitioner and is available on The Comprehensive R Archive Network (CRAN) as abglasso.

## The Bayesian DN

A fully Bayesian treatment of DNs remains unexplored and the novel methodology here aims to develop a simple yet highly accurate Bayesian DN estimation procedure. The novel contribution utilises the BAGLASSO as a launching point to separately estimate the components of the DN. The subsections that follow develop the framework for individual component estimation from a Bayesian viewpoint. Moreover, the framework has been develop for low *p* = 10 to moderate, *p* = 50 − 100, dimensions where *n* ≥ *p*.

### The Bayesian graphical lasso prior

Recall that the graphical lasso objective is maximising the penalized log-likelihood

$$\begin{array}{c} \underset{\Theta \in M^{+}}{\text{arg}\;\text{min}}l\left(\Theta \right), \end{array}$$

where

$$\begin{array}{c} l\left(\Theta \right)=\text{log}\left(\text{det}\Theta \right)-\text{trace}\left(\frac{S}{n}\Theta \right)-\rho {\left\|\Theta \right\|}_{1} \end{array}$$ (2)

and *M*^+^ is the space of positive definite matrices, **S** is the sample covariance matrix and *n* the sample size, respectively. More over, *ρ* ≥ 0 is the shrinkage parameter and **Θ** = (*θ*_*ij*_) is the precision matrix. The Bayesian connection to the graphical lasso problem is the maximum a posteriori (MAP) estimate, assuming a random sample from *N*_*p*_(***μ***, **Θ**^−1^), of the following

$$\begin{array}{c} p(\Theta \;|\lambda )=C^{-1}\prod_{i<j}\{\text{DE}(\theta_{ij}\;|\;\lambda )\}\prod_{i=1}^{p}\{\text{EXP}(\theta_{ii}\;|\;\lambda )\}\;\;\;\;\left(\Theta \in M^{+}\right). \end{array}$$ (3)

The prior distribution is given by the product of a double exponential (DE) with form *p*(*y*) = λ/2 exp(−λ|*y*|) for the off diagonal elements and an exponential (EXP) with form *p*(*y*) = λ exp(−λ*y*)1_*y* > 0_, otherwise. The value of **Θ** which maximizes the posterior density is the graphical lasso estimate in Eq (2) when *ρ* = λ/*n*. Within the Bayesian context λ is treated as the shrinkage parameter. The formulation and interpretations of the graphical lasso prior in Eq (3) have been studied in [26]. The aim therein is the development of varying regularization to infer block structures within the graphical models and efficiently estimating the maximum a posteriori of the corresponding posterior distribution. [16] make use of this prior formulation for the convenience (scale mixture of Gaussian formulation of the double exponential) in the development of their efficient block Gibbs sampler, in addition to allowing for the use of a gamma hyperprior on the shrinkage parameter λ for improved precision matrix estimation.

### Hierarchical representation

The Gibbs sampler for sampling the precision matrix **Θ** from the posterior distribution, defined below in Eq (5), associated with the prior in Eq (3), is constructed using a hierarchical representation of Eq (3). This particular hierarchical representation of the prior in Eq (3) is presented by [16], whom follow the same approach as in the development of the Gibbs sampler for the Bayesian lasso in [27]. The Gibbs sampler in [27] utilises the structure of the double exponential distribution as a scale mixture of Gaussians, assuming independence of the conditional double exponential priors [28, 29], in their hierarchical representation to simulate regression parameters from the desired posterior distribution. The positive definite constraint on **Θ** in Eq (3) implies that the Gaussian components for *θ*_*ij*_ (DE parameters) in the scale mixture formulation are no longer independent given the scale parameters. To address this issue, the hierarchical representation of the graphical lasso prior in Eq (3) is given by

$$\begin{array}{c} p\left(\theta \;|\;\tau ,\lambda \right)=C_{\tau }^{-1}\prod_{i<j}\{\frac{1}{\sqrt{2\pi \tau_{ij}}}\text{exp}\left(-\frac{\theta_{ij}^{2}}{2\tau_{ij}}\right)\}\prod_{i=1}^{p}\{\frac{\lambda }{2}\text{exp}\left(-\frac{\lambda }{2}\theta_{ii}\right)\}\;\;\;\;\left(\Theta \in M^{+}\right), \end{array}$$ (4)

where ***θ*** ≔ {*θ*_*ij*_}_*i*≤*j*_ is a vector of the upper triangular matrix entries of **Θ** and ***τ*** = {*τ*_*ij*_}_*i*<*j*_ the scale parameters. The normalising constant, *C*_**τ**_, has no closed-form solution. Obtaining the marginal distribution Eq (3), [16] propose a mixing density proportional to an exponential density with rate parameter λ^2^/2 and simple substitution circumvents the intractable normalising constant. Finally, the hierarchical representation in Eq (4) is used in the development of the block Gibbs sampler, available in the S1 File, with a target posterior distribution given by

$$p(\Theta ,\tau |Y,\lambda )\propto \text{det}\Theta^{\frac{n}{2}}\;\text{exp}\{-\text{trace}(\frac{1}{2}S\Theta )\}\prod_{i<j}\{\tau_{ij}^{-\frac{1}{2}}\text{exp}(-\frac{\theta_{ij}^{2}}{2\tau_{ij}})\text{exp}(-\frac{\lambda^{2}}{2}\tau_{ij})\}\times \prod_{i=1}^{p}\{\text{exp}(-\frac{\lambda }{2}\theta_{ii})\}(\Theta \in M^{+}).$$ (5)

### BAGLASSO

It is well known that the double exponential prior in Eq (3) may over-shrink (under-shrink) large (small) coefficients in **Θ**. The limitations within a regression context have been studied in [30–32] with alternative proposals. The BAGLASSO, Bayesian analog to the adaptive graphical lasso [33], exploits the framework and flexibility of the hierarchical representation in Eq (4) to address the aforementioned limitation. This extension serves to improve the accuracy of the precision matrix estimates obtained from the posterior in Eq (5) by allowing for different shrinkage parameters λ_*ij*_ for each corresponding off-diagonal precision matrix entry *θ*_*ij*_. Recall that the adaptive graphical lasso is given by

$$\begin{array}{c} \underset{\Theta \in M^{+}}{\text{arg}\;\text{min}}\;l\left(\Theta \right), \end{array}$$

where

$$\begin{array}{c} l\left(\Theta \right)=\text{log}\left(\text{det}\Theta \right)-\text{trace}\left(\frac{S}{n}\Theta \right)-\lambda \sum_{1\leq i\leq p}\sum_{1\leq j\leq p}\xi_{ij}\left|\theta_{ij}\right|, \end{array}$$ (6)

and $\xi_{ij}=1/|{\tilde{\theta }}_{ij}{|}^{\alpha }$ for *α* > 0 are the adaptive weights and the weight matrix (${\tilde{\theta }}_{ij}$) is the sample precision matrix.

The form of the Bayesian graphical lasso in Eq (3) enables the selection of an appropriate hyperprior on the shrinkage parameter λ, recall that *ρ* = λ/*n* in the Bayesian formulation of Eq (2). Adhering to the positive definite constraint on **Θ**, the prior normalising constant in Eq (3) when a single λ is applied to all elements in **Θ** can be obtained by applying the substitution $\tilde{\Theta }=\lambda \Theta$. Thereafter, a gamma prior λ ∼ GA(*r*, *s*) and corresponding conditional posterior λ ∼ GA(*r* + *p*(*p* + 1), *s* + ‖**Θ**‖_1_/2) can be obtained and sampled from. When allowing for individual λ_*ij*_’s for different off-diagonal *θ*_*ij*_’s, the normalising constant *C* will inevitably depend on λ_*ij*_. To address this a hierarchical formulation can be used to construct a set of prior distributions, serving as the the extension of the graphical lasso prior in Eq (3), for various λ_*ij*_ that mitigate the complications associated with posterior simulation due to the intractable normalising constant. This extension is the BAGLASSO and, assuming a random sample from *N*_*p*_(***μ***, **Θ**^−1^), is given by

$$\begin{array}{c} p\left(\Theta \;|\;{\left\{\lambda_{ij}\right\}}_{i\leq j}\right)=C_{{\left\{\lambda_{ij}\right\}}_{i\leq j}}^{-1}\prod_{i<j}\{\text{DE}(\theta_{ij}\;|\;\lambda_{ij})\}\prod_{i=1}^{p}\{\text{EXP}(\theta_{ii}\;|\;\frac{\lambda_{ii}}{2})\}\;\;\;\;\left(\Theta \in M^{+}\right), \end{array}$$

$$\begin{array}{c} p\left({\left\{\lambda_{ij}\right\}}_{i<j}\;|\;{\left\{\lambda_{ii}\right\}}_{i=1}^{p}\right)\propto C_{{\left\{\lambda_{ij}\right\}}_{i\leq j}}\prod_{i<j}\text{GA}\left(r,s\right). \end{array}$$ (7)

The normalising constant $C_{{\left\{\lambda_{ij}\right\}}_{i\leq j}}$ is intractable, as mentioned above, and the set ${\left\{\lambda_{ii}\right\}}_{i=1}^{p}$ are hyperparameters for the diagonal elements of **Θ**. Simple substitution yields that computation of λ_*ij*_ is simplified by circumventing the intractable normalising constant.

The BAGLASSO selects the amount of shrinkage λ_*ij*_ proportionally to the current value of *θ*_*ij*_. To see this [16], demonstrate that the conditional posterior, λ_*ij*_ | **Θ** ∼ GA(*r* + 1, |*θ*_*ij*_| + *s*), has an expected value that is inversely related to magnitude of *θ*_*ij*_. The data augmented block Gibbs sampler for the hierarchical representation in Eq (7) is the fundamental building block upon which the novel Bayesian DN is devised.

### Technicalities on conditional dependencies

Recall that the precision matrix directly determines the conditional dependence relations and structure of the undirected graphical model. Therefore, correctly estimating the precision matrices with sparse structures is essential to adequately gauge the conditional dependency relations between variables. The task to estimate the precision matrix for both *n* < *p* and *p* ≤ *n* remains challenging and regularization is often required [34–36]. A popular choice of prior for Bayesian posterior inference regarding network structure is the conjugate Wishart [37]. An alternative thresholding strategy is presented which is an adaption of the recommendation by [32]. In particular the conjugate Wishart W(3, *ϵ* **I**_*p*_) prior is used. The corresponding posterior is W(3 + *n*, (**S** + *ϵ* **I**_*p*_)^−1^), where *ϵ* = 0.001 and **I**_*p*_ a *p* dimensional identity matrix. The posterior samples are used to compute the posterior distribution of the *p* × *p* partial correlation matrix *P* ≔ {*ρ*_*ij*_}. The recommended strategy here suggests *θ*_*ij*_ ≠ 0 for *i* ≠ *j* if

$$\begin{array}{c} |E_{h}\left(\rho_{ij}\;|\;Y\right)|>\eta , \end{array}$$ (8)

where *η* may vary depending on the underlying graph structure. The Bayesian posterior thresholding recommendation by [16] claim that *θ*_*ij*_ ≠ 0 for *i* ≠ *j* if and only if

$$\begin{array}{c} \frac{{\tilde{\rho }}_{ij}}{E_{g}\left(\rho_{ij}\;|\;Y\right)}>\eta . \end{array}$$ (9)

Noting that ${\tilde{\rho }}_{ij}$ is the posterior sample mean estimate of the partial correlation under graphical lasso priors in Eq (3); *g* is the standard conjugate Wishart W(3, **I**_*p*_) and *h* the standard conjugate Wishart W(3, *ϵ* **I**_*p*_). Moreover, *η* ∈ [0, 1] with the lower and upper bounds resulting in a completely dense or sparse estimate, respectively.

**Fig 1:**
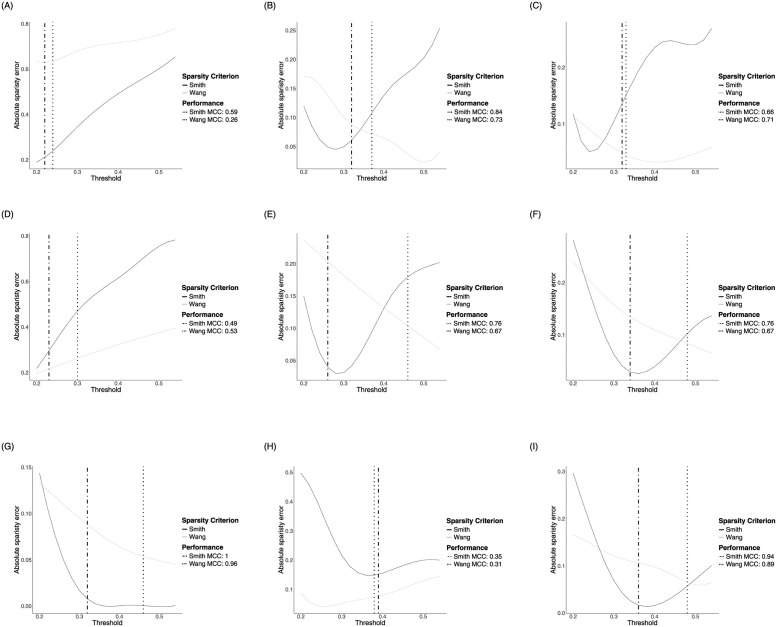
Optimal Bayesian sparsity threshold selection. The median of the absolute sparsity error and best performing MCC for various graph structures under varying thresholds for each Bayesian sparsity criterion in Eq (9) (dotted) and Eq (8) (dot-dash) for dimension *p* = 10. The best performing threshold is indicated by a vertical line with the accompanying MCC value displayed in the legend. (a) Model 1: AR(1). (b) Model 2: AR(2). (c) Model 3: at most 80% sparse. (d) Model 4: at most 40% sparse. (e) Model 5: scale-free. (f) Model 6: band. (g) Model 7: cluster. (h) Model 8: star. (i) Model 9: circle.

The original recommendation for *η* in Eq (9) is 0.5. The forthcoming synthetic data analysis section describes the simulation procedure, as well as, illustrates the performance of the Bayesian DN with regards to different graph structures, namely an AR(1), AR(2), sparse random, scale-free, band, cluster, star and circle. The goal here is to suggest a suitable sparsity threshold region under the varying graph structures for the recommended sparsity criterion in Eq (8). The Bayesian DN is applied across all graph structures with thresholds, *η*, in the range of 0.2 and 0.6 in increments of 0.02. The absolute sparsity error is computed for each graph structure for each Bayesian sparsity criterion in Eqs (8) and (9), respectively. The results are based on the median of 40 replications and the Matthews Correlation Coefficient (MCC), see [38], is used to determine the best performing threshold. Fig (1a)–(1i) display the optimal threshold, based on the top performing MCC, for each graph structure and Bayesian sparsity criterion for *p* = 10. The optimal threshold plots for *p* = 30 and *p* = 100 are available in the S1 File. The optimal threshold based on Eq (8), η*, for the Bayesian DN is, in most cases, in the neighborhood of the minimum absolute sparsity error and in the region of η* ∈ {0.2 − 0.4}. Both Bayesian sparsity criterion candidates perform comparably well noting, however, that Eq (8) requires less computation.

## Synthetic data analysis

The synthetic experiment is designed to test the parameter estimation and graphical structure determination of the DN estimation for both the novel Bayesian approach (referred to as ‘B-net’) and the iterative shrinkage-thresholding estimator (referred to as ‘D-net’) from [24]. The iterative shrinkage-thresholding estimator uses the lasso penalty and Bayesian Information Criterion (BIC) for model estimation and selection, respectively. For all simulations, the assumption is that the observations, $x_{1},x_{2},\ldots ,x_{n_{1}}$ and $y_{1},y_{2},\ldots ,y_{n_{2}}$ are generated from a Gaussian *N*_*p*_(0, **Σ**_1_) and *N*_*p*_(0, **Σ**_2_) respectively. The true DN is

$$\begin{array}{c} \Delta =\Sigma_{2}^{-1}-\Sigma_{1}^{-1}, \end{array}$$

where the true precision matrices are $\Theta_{1}=\Sigma_{1}^{-1}$ and $\Theta_{2}=\Sigma_{2}^{-1}$. The Bayesian DN applies the BAGLASSO Eq (7) to each sample, i.e. separately estimates the precision matrices. Furthermore, for excellent performance set *r* = 10^−2^ and *s* = 10^−6^, see S1 File for more details, for the hyperparameters of the prior distributions of λ_*ij*_ for *i* < *j* and λ_*ii*_ = 1 for *i* = 1, …, *p*. The iterative shrinkage-thresholding approach jointly estimates the precision matrices for Eq (1). The following 9 graphical structure variations are considered—where the structure of each is applied to each component in the DN’s composition to achieve the desired structure in the DN itself—in the simulation:

- *structure 1*: An AR(1) model.
  - *Component 1*: *θ*_*ij*_ = 0.7^|*i*−*j*|^.
  - *Component 2*: *θ*_*ij*_ = 0.75^|*i*−*j*|^.
- *structure 2*: An AR(2) model.
  - *Component 1*: *θ*_*ii*_ = 0.1, *θ*_*i*,*i*−1_ = *θ*_*i*−1,*i*_ = 0.05 and *θ*_*i*,*i*−2_ = *θ*_*i*−2,*i*_ = 0.025.
  - *Component 2*: *θ*_*ii*_ = 1,*θ*_*i*,*i*−1_ = *θ*_*i*−1,*i*_ = 0.5 and *θ*_*i*,*i*−2_ = *θ*_*i*−2, *i*_ = 0.25.
- *structure 3*: A sparse random model where both components have approximately up to 80% off-diagonal elements set to zero.
- *structure 4*: A moderately sparse random model where both components have approximately up to 40% off-diagonal elements set to zero.
- *structure 5*: A scale-free model where the second component is a scalar multiple of the first.
- *structure 6*: A band or diagonal model.
  - *Component 1*: *θ*_*ii*_ = 1, *θ*_*ij*_ = 0.2 for 1 ≤ *i* ≠ *j* ≤ *p*/2, *θ*_*ij*_ = 0.5 for *p*/2 + 1 ≤ *i* ≠ *j* ≤ *p* and *θ*_*ij*_ = 0 otherwise.
  - *Component 2*: *θ*_*ii*_ = 1, *θ*_*ij*_ = 0.7 for 1 ≤ *i* ≠ *j* ≤ *p*/2, *θ*_*ij*_ = 0.9 for *p*/2 + 1 ≤ *i* ≠ *j* ≤ *p* and *θ*_*ij*_ = 0 otherwise.
- *structure 7*: A cluster model containing two disjoint groups.
  - *Component 1*: *θ*_*ii*_ = 1, *θ*_*ij*_ = 0.5 for 1 ≤ *i* ≠ *j* ≤ *p*/2, *θ*_*ij*_ = 0.5 for *p*/2 + 1 ≤ *i* ≠ *j* ≤ *p* and *θ*_*ij*_ = 0 otherwise.
  - *Component 2*: *θ*_*ii*_ = 1, *θ*_*ij*_ = 0.9 for 1 ≤ *i* ≠ *j* ≤ *p*/2, *θ*_*ij*_ = 0.9 for *p*/2 + 1 ≤ *i* ≠ *j* ≤ *p* and *θ*_*ij*_ = 0 otherwise.
- *structure 8*: A star model with every node connected to the first node.
  - *Component 1*: *θ*_*ii*_ = 1, *θ*_1,*i*_ = *θ*_*i*,1_ = 0.1 and *θ*_*i*,*j*_ = 0. otherwise.
  - *Component 2*: *θ*_*ii*_ = 1, *θ*_1,*i*_ = *θ*_*i*,1_ = 2.1 and *θ*_*i*,*j*_ = 0. otherwise.
- *structure 9*: A circular model.
  - *Component 1*: *θ*_*ii*_ = 2, *θ*_*i*,*i*−1_ = *θ*_*i*−1,*i*_ = 1 and *θ*_1,*p*_ = *θ*_*p*,1_ = 0.45.
  - *Component 2*: *θ*_*ii*_ = 4, *θ*_*i*,*i*−1_ = *θ*_*i*−1,*i*_ = 2 and *θ*_1,*p*_ = *θ*_*p*,1_ = 0.95.

The sample sizes and dimensions for each model are *n*_1_ = *n*_2_ ∈ {50, 100, 200} and *p*_1_ = *p*_2_ ∈ {10, 30, 100}, respectively. The Bayesian estimates are based on 10000 Monte Carlo iterations after 5000 burn-in iterations. To assess the performance of DN matrix estimation, six loss functions are considered and defined in Table 1, where *p* denotes the dimension and *γ*_*i*_ the *i*^*th*^ eigenvalue, respectively. Notice that some loss functions utilise the true DN matrix and its estimates, while others utilise the eigenvalues and their respective estimates. Table 2 reports the median of L1, L2, EL1, EL2, MAXEL1 and MINEL1 for *p* = 10, 30, 100 in structures 1−9 based on 40 replications. For each scenario, the best performing measure is boldfaced.

**Table 1:** Loss functions used in the synthetic data analysis to assess the numerical accuracy of the B-net and D-net estimates.

Measure	Loss function	Abbreviation
Matrix *L*_1_-norm	$\|\hat{\Delta }{-\Delta \|}_{1}=max_{1\leq j\leq p}\sum_{i=1}^{p}\left|{\hat{\Delta }}_{ij}-\Delta_{ij}\right|$	L1
Frobenius loss	$\|\hat{\Delta }{-\Delta \|}_{F}$ , where ${\left\|A\right\|}_{F}^{2}=trace\left(AA^{⊤}\right)$	L2
*L*_1_ eigenvalue loss	$\sum_{i=1}^{p}\left|{\hat{\gamma }}_{i}-\gamma_{i}\right|/p$	EL1
*L*_2_ eigenvalue loss	$\sum_{i=1}^{p}{\left({\hat{\gamma }}_{i}-\gamma_{i}\right)}^{2}/p$	EL2
*L*_1_ loss on the largest eigenvalue	$|{\hat{\gamma }}_{max}-\gamma_{max}|$	MAXEL1
*L*_1_ loss on the smallest eigenvalue	$|{\hat{\gamma }}_{min}-\gamma_{min}|$	MINEL1

**Table 2:** Synthetic study median loss results.

	AR(1)		AR(2)		S80		S40		SF		Band		Cluster		Star		Circle	
	B-net	D-net	B-net	D-net	B-net	D-net	B-net	D-net	B-net	D-net	B-net	D-net	B-net	D-net	B-net	D-net	B-net	D-net
p = 10																		
L1	1.04	**0.64**	**1.13**	1.49	**2.26**	3.41	**6.03**	7.56	**2.24**	2.79	**1**	1.21	**0.85**	1.68	18.47	**18**	2.73	**2**
L2	0.91	**0.64**	**1.41**	2.09	**2.21**	3.8	**6.09**	7.45	**2.14**	2.22	**1.39**	1.91	**1.05**	2.48	**8.73**	**8.49**	3.87	4.3
EL1	0.14	**0.1**	**0.24**	0.53	**0.23**	0.61	**0.63**	1.12	**0.11**	0.48	**0.22**	0.43	**0.25**	0.58	**1.19**	1.2	**0.42**	1.23
EL2	0.03	**0.03**	**0.09**	0.35	**0.1**	0.47	**0.47**	1.73	**0.02**	0.49	**0.07**	0.21	**0.1**	0.52	**6.02**	7.2	**0.27**	1.83
MAXEL1	**0.17**	0.51	**0.49**	1.07	**0.2**	0.72	**0.7**	1.57	**0.15**	1.37	**0.44**	0.55	**0.64**	1.42	**5.45**	6	**0.58**	1.91
MINEL1	0.28	**0.06**	**0.09**	0.45	**0.16**	0.67	**0.9**	1.09	**0.15**	1.37	**0.44**	0.49	0.36	**0.22**	**5.52**	6	**0.6**	1.91
p = 30																		
L1	1.86	**1.27**	**1.04**	1.52	**10.48**	13.66	**25.16**	30.8	**5.98**	5.21	**0.97**	1	5.89	**5.65**	58.07	**58**	**2.72**	2.06
L2	1.98	**1.35**	**1.93**	3.82	**13.11**	17.03	**26.02**	32	3.77	**2.87**	**2.21**	3.67	8.39	**8.2**	15.26	**15.23**	**4.96**	7.65
EL1	0.16	**0.12**	**0.17**	0.58	**0.77**	2.4	**1.75**	4.67	**0.22**	0.31	**0.22**	0.59	**0.72**	0.74	**0.71**	0.72	**0.15**	1.25
EL2	**0.04**	0.06	**0.04**	0.43	**0.75**	7.36	**3.93**	28.49	**0.1**	0.27	**0.07**	0.42	**2.1**	2.21	**7.29**	7.73	**0.04**	1.92
MAXEL1	**0.56**	1.07	**0.22**	1.2	**1.04**	3.41	**2.84**	8.34	**1.05**	1.62	**0.49**	0.81	**5.41**	5.55	**10.45**	10.77	**0.64**	1.93
MINEL1	0.38	**0.14**	**0.16**	0.53	**1.14**	3.4	**3.03**	8.23	**0.85**	1.62	**0.49**	0.81	**0.19**	0.35	**10.45**	10.77	**0.64**	1.93
p = 100																		
L1	2.11	**1.33**	**1.02**	1.35	**44.18**	46.52	**91.89**	96.26	**7.04**	**7.03**	0.97	1	19.88	**19.61**	**198.16**	198.32	2.81	**2.03**
L2	3.36	**2.62**	**3.1**	7.07	**58.3**	62.13	**103.75**	108.41	5.56	**3.88**	**3.87**	7.04	28.2	**28**	**28.17**	28.19	9.17	**14.09**
EL1	0.14	**0.13**	**0.15**	0.63	**3.37**	5.17	**5.04**	9.08	**0.11**	0.23	**0.27**	0.63	**0.78**	0.78	**0.4**	0.4	**0.18**	1.27
EL2	**0.03**	0.07	**0.03**	0.5	**14.36**	36.49	**33.86**	114.22	**0.03**	0.15	**0.1**	0.49	**7.69**	7.83	**7.78**	7.83	**0.06**	1.98
MAXEL1	**0.46**	1.29	**0.07**	1.35	**6.22**	9.97	**10.41**	18.07	**0.1**	1.77	**0.53**	1	**19.4**	19.58	**19.72**	19.79	**0.83**	1.98
MINEL1	0.48	**0.17**	**0.31**	0.67	**6.18**	10.04	**9.86**	17.81	**0.1**	1.77	**0.53**	1	**0.2**	0.38	**19.72**	19.79	**0.83**	1.98

Summary of L1, L2, EL1, EL2, MAXEL1 and MINEL1 for an AR(1), AR(2), sparse random, scale-free, band, cluster, star and circle graphical model. The median loss values reported here are based on 40 replications for both the B-net and D-net estimators. The best performing values are boldfaced.

The eigenvalue based loss functions are designed to investigate the extremes of the eigenvalue spectrum. In particular, the MAXEL1 loss function highlights which estimator is favourable in a principal component setting, [39]. A couple of observations are worth noting from Tables 2 and 3. First, the D-net estimator performs better with the AR(1) structure. Second, the B-net estimator performs exceptionally well in remaining structures. Third, the standard errors for both DN estimation techniques remain relatively consistent throughout the dimension spectrum considered, noting that the D-net estimator yields, in general, better results. This may be due to the fact that the best performing tuning parameter in the solution path leads to highly sparse estimates. The B-net estimation procedure inherits the utilisation of multiple penalty parameters in the precision matrix estimation, leading to robust estimation of the precision matrices.

**Table 3:** Synthetic study standard error loss results.

	AR(1)		AR(2)		S80		S40		SF		Band		Cluster		Star		Circle	
	B-net	D-net	B-net	D-net	B-net	D-net	B-net	D-net	B-net	D-net	B-net	D-net	B-net	D-net	B-net	D-net	B-net	D-net
p = 10																		
L1	0.19	0.05	0.14	**0.09**	**0.72**	1.16	**1.37**	2.42	0.16	**0.01**	0.29	**0.19**	0.33	**0.12**	0.79	**0.12**	0.69	**0.12**
L2	0.15	**0.03**	0.15	**0.04**	**0.78**	1.02	**1.07**	2.11	0.09	**0.01**	0.37	**0.15**	0.37	**0.05**	0.42	**0.06**	0.89	**0.01**
EL1	0.02	**0.01**	0.07	**0.04**	**0.12**	0.24	**0.19**	0.77	0.02	**0.01**	**0.09**	0.1	0.08	**0.03**	0.06	**0.02**	0.14	**0.03**
EL2	**0.01**	**0.01**	**0.05**	**0.05**	**0.11**	0.41	**0.39**	3.51	0.01	**0.01**	**0.06**	0.1	0.09	**0.04**	0.8	**0.29**	0.17	**0.1**
MAXEL1	0.11	**0.01**	0.25	**0.08**	**0.27**	0.5	**0.57**	1.28	0.08	**0.01**	**0.17**	0.18	0.3	**0.08**	0.4	**0.12**	0.48	**0.1**
MINEL1	0.13	**0.04**	0.1	**0.08**	**0.26**	0.57	**0.64**	1.46	0.08	**0.01**	**0.17**	0.22	0.12	**0.09**	0.38	**0.12**	0.5	0.1
p = 30																		
L1	0.22	**0.01**	0.14	**0.11**	1.45	**1.25**	**1.63**	2.28	0.11	**0.01**	0.16	**0.04**	0.08	**0.04**	0.22	**0.03**	0.5	0.05
L2	0.2	**0.01**	0.14	**0.01**	**1.03**	1.23	**1.07**	1.23	0.05	**0.01**	0.27	**0.2**	0.04	**0.01**	0.06	**0.01**	1.07	**0.01**
EL1	0.02	**0.01**	0.03	**0.02**	**0.2**	0.25	**0.18**	0.3	**0.01**	**0.01**	**0.04**	**0.04**	**0.01**	**0.01**	**0.01**	**0.01**	0.05	**0.01**
EL2	**0.01**	**0.01**	**0.01**	0.03	**0.39**	1.58	**0.86**	4.6	**0.01**	**0.01**	**0.03**	0.07	0.03	0.02	0.15	**0.05**	0.05	**0.03**
MAXEL1	0.15	**0.02**	0.12	**0.06**	**0.61**	1.31	**0.76**	1.58	0.06	**0.01**	**0.1**	0.11	0.04	**0.03**	0.11	**0.04**	0.28	**0.04**
MINEL1	0.16	**0.02**	0.07	**0.05**	**0.61**	1.37	**0.83**	1.6	0.03	**0.01**	**0.11**	0.12	0.05	**0.03**	0.11	**0.04**	0.28	**0.04**
p = 100																		
L1	0.24	**0.01**	0.12	**0.02**	**2.26**	2.85	**2.49**	4.02	0.1	**0.01**	**0.01**	**0.01**	0.03	**0.01**	**0.07**	0.36	0.19	**0.01**
L2	0.2	**0.01**	0.05	**0.01**	1.17	**1.1**	1.85	**1.71**	**0.01**	**0.01**	0.02	**0.01**	0.02	**0.01**	**0.01**	0.05	1.31	**0.01**
EL1	**0.01**	**0.01**	**0.01**	**0.01**	0.17	**0.11**	0.22	**0.16**	**0.01**	**0.01**	**0.01**	**0.01**	**0.01**	**0.01**	**0.01**	**0.01**	0.04	**0.01**
EL2	**0.01**	**0.01**	**0.01**	**0.01**	**1.45**	1.56	**2.98**	3.84	**0.01**	**0.01**	**0.01**	**0.01**	**0.01**	**0.01**	**0.06**	0.08	**0.01**	**0.01**
MAXEL1	0.19	**0.01**	0.07	**0.01**	**0.75**	0.89	**1.08**	1.32	0.03	**0.01**	**0.03**	**0.03**	**0.01**	**0.01**	**0.07**	0.1	0.14	**0.01**
MINEL1	0.12	**0.01**	0.05	**0.01**	**0.6**	0.89	**0.89**	1.52	0.03	**0.01**	**0.03**	**0.03**	**0.01**	**0.01**	**0.07**	0.1	0.14	**0.01**

Summary of L1, L2, EL1, EL2, MAXEL1 and MINEL1 for an AR(1), AR(2), sparse random, scale-free, band, cluster, star and circle graphical model. The median standard errors reported here are based on 40 replications for both the B-net and D-net estimators. The best performing values are boldfaced.

To assess the performance on graphical structure determination, the specificity, sensitivity, false negative rate, f1 score and the MCCs are computed and defined in Table 4. Noting that, TP, TN, FP and FN denote the number of true positives, true negatives, false positives and false negatives, respectively. Values of specificity, sensitivity, f1-score and MCC closer to one, imply better classification performance. The closer the values of false negative rate are to zero the better. Further insights on the performance metrics are discussed in [40]. The sparsity for the B-net estimator is determined by the thresholding rule in Eq (8) and the thresholds, *η*, associated with the MCC values in Fig (1a)–(1i). Similarly, the best performing tuning parameter in the solution path of the D-net algorithm determines the sparsity of the estimator. The median performance scores, based on 40 repetitions, for each graphical structure is presented in Table 5. The main diagonals of the adjacency matrices were not included in the scoring.

**Table 4:** Performance measures used to assess classification accuracy of the B-net and D-net graphical models estimates.

Measure	Performance function	Abbreviation
Specificity	$\frac{\text{TN}}{\text{TN}+\text{FP}}$	SP
Sensitivity	$\frac{\text{TP}}{\text{TP}+\text{FN}}$	SE
False negative rate	$\frac{FP}{FP+TN}$	FNR
F1-score	$\frac{TP}{TP+\frac{1}{2}\left(FP+FN\right)}$	F1
Matthews Correlation Coefficient	$\frac{\text{TP}\times \text{TN}-\text{FP}\times \text{FN}}{\sqrt{\left(\text{TP}+\text{FP})(\text{TP}+\text{FN})(\text{TN}+\text{FP})(\text{TN}+\text{FN}\right)}}$	MCC

**Table 5:** Synthetic study median performance results.

	AR(1)		AR(2)		S80		S40		SF		Band		Cluster		Star		Circle	
	B-net	D-net	B-net	D-net	B-net	D-net	B-net	D-net	B-net	D-net	B-net	D-net	B-net	D-net	B-net	D-net	B-net	D-net
p = 10																		
SE	**0.72**	0.11	**0.82**	0.21	**0.6**	0.53	**0.65**	0.36	**0.89**	NA	**0.89**	0.44	**1**	0.25	**0.22**	0.11	**0.9**	0
SP	**1**	**1**	**0.97**	0.7	**0.89**	0.82	0.87	0.94	**0.9**	NA	**0.95**	0.93	**1**	0.87	**0.8**	1	0.95	**0.98**
PR	**0.87**	0.5	**0.3**	0.12	**0.22**	**0.22**	0.72	0.56	**0.18**	NA	**0.16**	0.11	**0.4**	0.16	**0.06**	0.02	**0.2**	0
MC	**0.45**	0.11	**0.76**	-0.07	**0.54**	0.39	**0.43**	0.24	**0.71**	NA	**0.83**	0.41	**1**	0.14	**0.11**	0.3	**0.88**	-0.07
F1	**0.84**	0.2	**0.84**	0.24	**0.67**	0.56	**0.77**	0.51	**0.76**	NA	**0.86**	0.4	**1**	0.34	**0.25**	0.2	**0.9**	0
FNR	**0.28**	0.89	**0.18**	0.79	**0.4**	0.47	**0.35**	0.64	**0.11**	NA	**0.11**	0.56	**0**	0.75	**0.78**	0.89	**0.1**	1
p = 30																		
SE	**0.31**	0.01	**0.75**	0.04	0.23	0.08	**0.37**	0.03	**0.79**	NA	**0.9**	0.24	**0.89**	0.02	**0.03**	0.02	**0.97**	0
SP	0.97	**1**	**0.99**	0.93	0.98	**0.99**	0.82	**1**	**0.92**	NA	**1**	**1**	0.99	0.98	**1**	**1**	**1**	0.99
MC	**0.17**	0.02	**0.8**	-0.04	**0.35**	0.16	**0.16**	0.05	**0.52**	NA	**0.92**	0.46	**0.89**	-0.02	0.06	**0.08**	**0.95**	-0.03
F1	**0.48**	0.01	**0.82**	0.04	**0.36**	0.14	**0.53**	0.06	**0.53**	NA	**0.93**	0.38	**0.93**	0.04	**0.06**	0.03	**0.95**	0
FNR	**0.69**	0.99	**0.25**	0.96	**0.77**	0.92	**0.63**	0.97	**0.21**	NA	**0.1**	0.76	**0.11**	0.98	**0.97**	0.98	**0.03**	1
p = 100																		
SE	**0.21**	0	**0.73**	0	**0.04**	0.01	**0.15**	0	**0.49**	NA	**0.95**	0.05	**0.79**	0	0.02	**0.12**	**0.99**	0
SP	**0.99**	1	**1**	**1**	**1**	**1**	0.92	**1**	**0.98**	NA	**1**	**1**	**1**	**1**	**1**	**1**	**1**	**1**
MC	**0.33**	0.01	**0.82**	0	**0.14**	0.05	**0.07**	0.01	**0.39**	NA	**0.95**	0.22	**0.81**	0	0.08	**0.35**	**0.98**	-0.01
F1	**0.34**	0	**0.82**	0	**0.08**	0.01	**0.25**	0	**0.4**	NA	**0.95**	0.1	**0.88**	0	0.04	**0.22**	**0.98**	0
FNR	**0.79**	1	**0.27**	1	**0.96**	0.99	**0.85**	1	**0.51**	NA	**0.05**	0.95	**0.21**	1	0.98	**0.88**	**0.01**	1

Summary of SE, SP, F1, MC and for an AR(1), AR(2), sparse random, scale-free, band, cluster, star and circle graphical model. The median performance scores reported here are based on 40 replications for both the B-net and D-net estimators. The best performing values are bold-faced and scores that were not attainable due to single class classification are encoded as NA.

**Fig 2:**
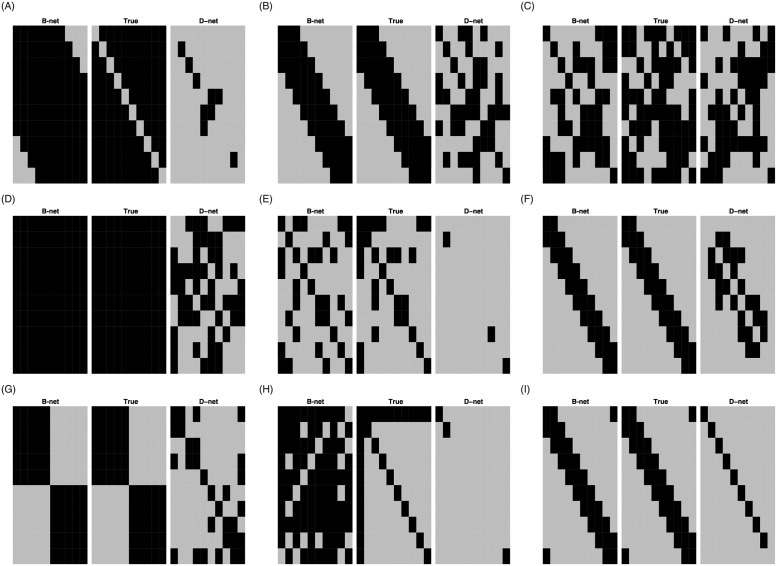
DN adjacency matrix heatmaps. Comparison of the true DN, B-net and D-net adjacency matrices for an AR(1), AR(2), sparse random, scale-free, band, cluster, star and circle graphical model and *p* = 10. (a) Model 1: AR(1). (b) Model 2: AR(2). (c) Model 3: at most 80% sparse. (d) Model 4: at most 40% sparse. (e) Model 5: scale-free. (f) Model 6: band. (g) Model 7: cluster. (h) Model 8: star. (f) Model 9: circle.

**Fig 3:**
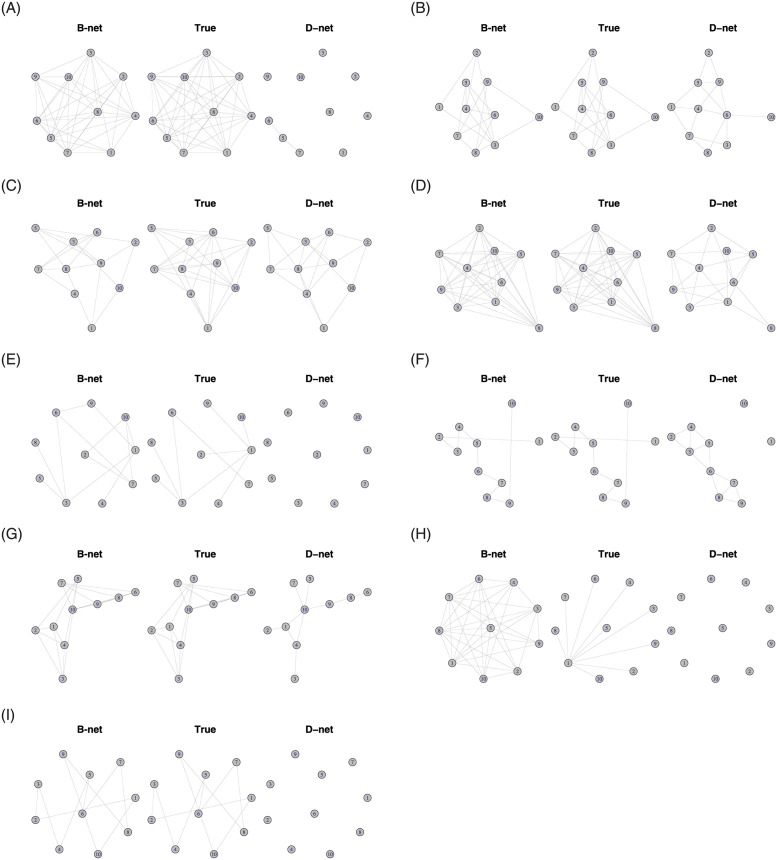
DN adjacency matrix graphical models. Comparison of the true DN, B-net and D-net graphical structure estimates for an AR(1), AR(2), sparse random, scale-free, band, cluster, star and circle graphical model and *p* = 10. (a) Model 1: AR(1). (b) Model 2: AR(2). (c) Model 3: at most 80% sparse. (d) Model 4: at most 40% sparse. (e) Model 5: scale-free. (f) Model 6: band. (g) Model 7: cluster. (h) Model 8: star. (i) Model 9: circle.

The B-net estimator generally outperforms the D-net estimator across all models and all dimensions according to the MCC, f1-score, sensitivity and false negative rate, with the exception of the star case for *p* = 100. Fig (3a)–(3i) display the true and inferred undirected DN graphs for both the B-net and D-net estimators for *p* = 10; higher dimensions are available in the S1 File. Lastly, Fig (2a)–(2i) display the true and inferred adjacency matrices for *p* = 10. Both Figs 2 and 3 visually demonstrate the superiority of the B-net estimator.

## Real data analysis

This section focuses on applying the novel Bayesian DN estimator, B-net, as well as the terative shrinkage-thresholding estimator, D-net, to the spambase dataset, available at https://archive.ics.uci.edu/ml/datasets/spambase to investigate changes in DN structure between spam and non-spam data. In addition, the B-net estimator is applied to South African COVID-19 data, obtained from https://www.nicd.ac.za/diseases-a-z-index/disease-index-covid-19/surveillance-reports/, https://ourworldindata.org/coronavirus/country/south-africa and https://mediahack.co.za/datastories/coronavirus to investigate the change in DN structure between various phases of the pandemic.

### Spam data

The objective here is to compare the B-net and D-net graphical model estimates of the spam and non-spam emails. The dataset consists of 1813 spam emails and 2788 non-spam emails. The attributes include, amongst others, the average length of uninterrupted sequences of capital letters; total number of capital letters in the e-mail; an indicator denoting whether the e-mail was considered spam or not, in this study.

**Fig 4:**
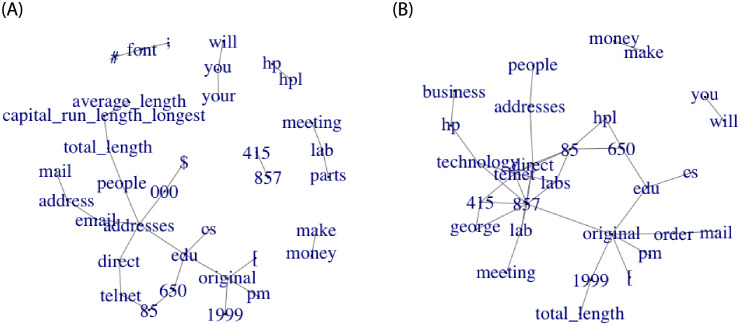
A comparison of the D-net and B-net DN estimates for the spam emails dataset. (a) The Bayesian DN for the spam emails dataset. (b) The iterative-shrinkage DN for the spam emails dataset.

Following the approach of [24], the data is standardised using a non-paranormal transformation in order to satisfy the Gaussian assumption. The B-net estimates are based on 10000 iterations of the Monte Carlo sampler after 5000 burn-in iterations. Fig 4 illustrates the difference between the B-net and D-net estimates. Both estimators indicate the presence of several common hub features namely, ‘edu’, ‘original’, ‘direct’, ‘lab’, ‘telnet’ and ‘addresses’. It is clear from both panes that the state of the covariance matrix structure between the spam and non-spam emails may very well be different. Furthermore, given that Hewlett-Packard Labs donated the data, words such as ‘telnet’ and ‘hp’ appear more often in the non-spam emails and can be used to distinguish between spam and non-spam emails.

### South African COVID-19 data

The 2019 novel coronavirus (COVID-19) has affected more than 180 countries around the world, including South Africa. The current body of knowledge boasts a wealth of statistical literature that aims at empowering researchers to study and alleviate the impact of the disease, see for example [41]. Understanding the interaction of key metrics and attributes between various phases, cycles or waves of the pandemic may prove to be invaluable in strategic planning and prevention. The goal here, is to use the Bayesian DN, B-net, to illustrate that the interactivity of key daily metrics between suspected homogeneous and heterogeneous phases within the pandemic life cycle is ever changing. In particular, the B-net is used to model the interactivity of daily metrics between the first two peaks or waves; the first wave and the following plateau and finally the difference between the first and second post wave plateaus. The data consists of 446 observations from the 7^th^ of February 2020 to the 27^th^ of April 2021. The daily metrics include, deaths; performed tests; positive test rate; active cases; tests per active case; recoveries; hospital admissions; hospital discharges; ICU admissions and the number of ventilated patients. It should be noted that no sensitive patient information is used, however, the interested reader is referred to [42] for a detailed treatment and framework for dealing with and sanitizing medical data containing sensitive patient information. Due to the irregularities in data capturing and publishing, a seven day moving average is applied across all daily metrics. The data is standardised using a non-paranormal transformation in order to satisfy the Gaussian assumption. The B-net is applied to the data using 10000 iterations of the Monte Carlo sampler after 5000 burn-in iterations.

**Fig 5:**
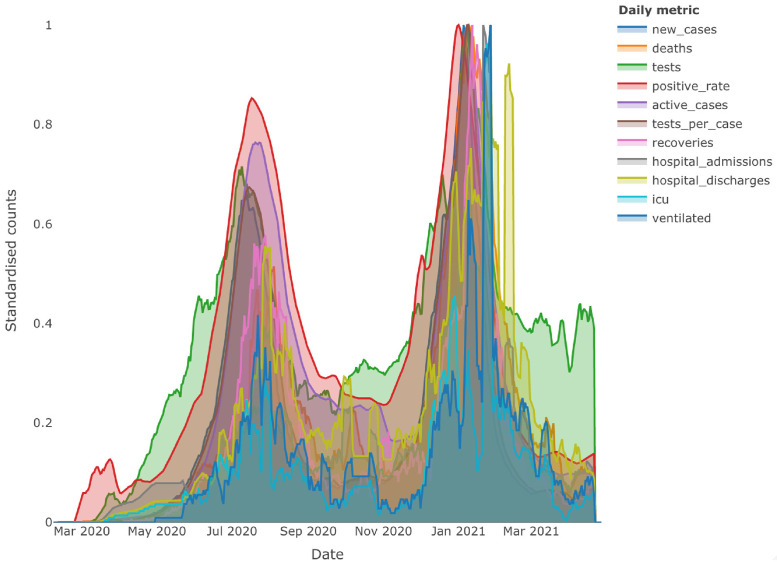
South African COVID-19 daily metrics over time. 7-day moving average filled area line plots with standardised counts for daily new cases; deaths; tests; positive test rate; active cases; tests per active case; recoveries; hospital admissions; hospital discharges; ICU admissions and ventilated patients.

**Fig 6:**
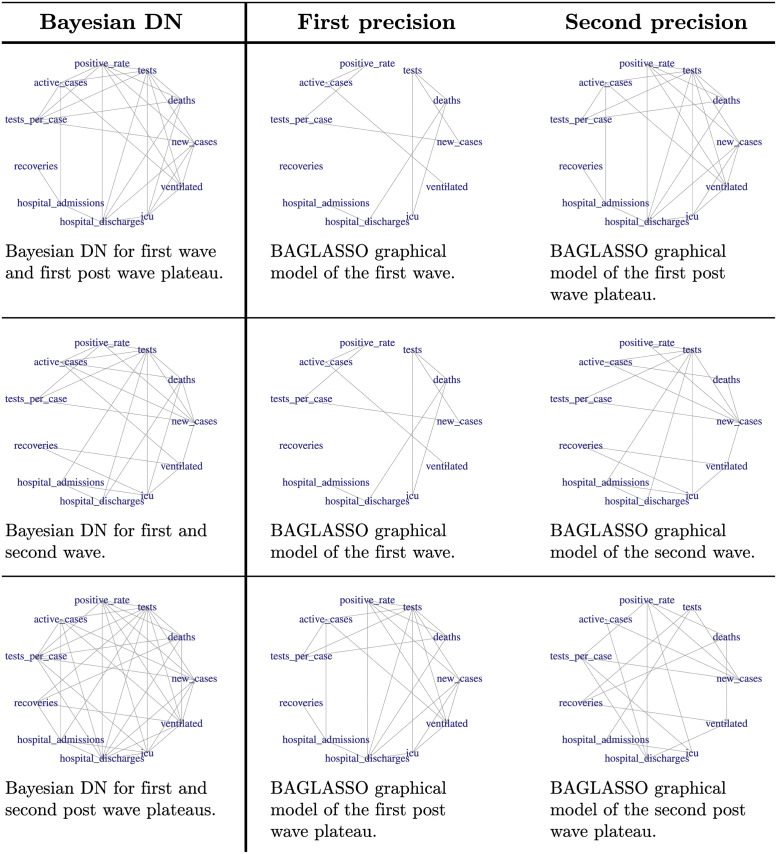
Bayesian DN estimates of South African COVID-19 data. The Bayesian DN and corresponding BAGLASSO graphical models between the first two waves; the first wave and the following plateau and finally the difference between the first and second post wave plateaus. The *p*−*values* from the Box’s M-test for homogeneity of covariance matrices between the contributing precision matrices were all less than 0.001 [43].

Fig 5 highlights the temporal nature of the pandemic between suspected homogeneous and heterogeneous phases. In other words, comparing the cyclical behaviour of individual daily metrics may seem clearly distinctive over time; a peak or wave is always followed by a plateau. Furthermore, extrapolation of the temporal behaviour of individual daily metrics may incorrectly allude to distinct multi modality of multiple daily metrics. Upon observing multiple metrics simultaneously, the crisp group-wise multi modality diminishes rather rapidly. The figures in Fig 6 illustrate the higher proportions of hub features present in the DNs. Interestingly, the Bayesian DN provides insight to the change in interaction between daily metrics between perceived homogeneous pandemic phases, that is comparisons between the two peaks and two post-peak plateaus. This change in behaviour could be as a result of the change in population adherence to public sanitation awareness; weather conditions; virus mutations or complacency of over time.

## Discussion

The Bayesian differential network estimator is the first of its kind which utilises the excellent graphical structure determination and matrix estimation of the Bayesian graphical lasso [16]. In comparison with the state of the art iterative shrinkage-thresholding approach, the Bayesian differential network offers MCMC outputs that allow the user to gain deeper insight and inference in the estimation procedure. The numerical accuracy of the Bayesian differential network is, in general, superior to that of the iterative shrinkage-thresholding estimator. Moreover, the Bayesian proposal captures both sparse and dense precision matrix patterns in some well-known graphical structures more accurately. The latter being a result of the Wishart prior’s ability to accommodate the variability and adjustment to the data. Furthermore, the thresholding technique for sparse estimation is designed such that it accounts for the effect of prior allocation through the posterior expectation.

The graphical structure learning is a crucial component of the Bayesian differential network estimator. The ad hoc approach provided in Eq (8) suggests a suitable sparsity threshold under varying graph structures. The Bayesian differential network also provides key insights to changes in the interactive behaviour of real data metrics ranging from filtering spam emails to COVID-19 life cycles. For high-dimensional data, the block Gibbs sampler may be adjusted to incorporate the singular normal distribution presented in [44] in the hierarchical representation Eq (7). Furthermore, research on simultaneous Bayesian estimation and optimisation of both $\Sigma_{1}^{-1}$ and $\Sigma_{2}^{-1}$ in the construction of the differential network is underway.

## Supporting information

S1 File Supplementary material. Contains a block Gibbs sampler, as well as, additional optimal threshold; adjacency heatmaps and graphical network figures for dimensions *p* = 30 and *p* = 100.
(PDF)

## Abbreviations

### List of abbreviations

ADMM: Alternating direction method of multipliers
BAGLASSO: Bayesian adaptive graphical lasso
B-net: Bayesian differential network
CRAN: Comprehensive R archive network
DE: Double exponential
DN: Differential network
D-net: Iterative shrinkage-thresholding differential network
EL1: L1 eigenvalue loss
EL2: Frobenius eigenvalue loss
EXP: Exponential
FNR: False negative rate
FN: False negative
FP: False positive
F1: F1-score
GCL: Graphical cooperative lasso
GGM: Gaussian graphical model
IGL: Intertwined graphical lasso
L1: L1 loss
L2: Frobenius loss
MAP: Maximum a posteriori
MAXEL1: L1 loss on the largest eigenvalue
MINEL2: L1 loss on the smallest eigenvalue
MCC: Matthews correlation coefficient
SE: Sensitivity
SP: Specificity
TN: True negative
TP: True positive

### List of symbols

${\tilde{\theta }}_{ij}$: Element *ij* of the adaptive graphical lasso sample precision matrix
G: Graphical model
E: Set of edges of a graphical model G
V: Set of nodes of a graphical model G
$M^{+}$: Space of positive definite matrices
**Σ**: Gaussian covariance matrix
*n*: Sample size
***P***: Partial correlation matrix
*r*: Gamma shape parameter
*s*: Gamma scale parameter
**S**: Sample covariance matrix
**Δ**: Differential network
*η*: Bayesian sparsity criterion threshold
**Θ**: Gaussian precision matrix
*θ*_*ij*_: Element *ij* of the precision matrix **Θ**
***μ***: Gaussian mean vector
*ξ*: Weights of the adaptive graphical lasso
*ρ*: Lasso penalty parameter
*τ*: Scale mixture of Gaussian scale parameter
