# Supplementary material for: Empowering differential networks using Bayesian analysis
Source: PLoS One. 2022 Jan 25;17(1):e0261193. doi: 10.1371/journal.pone.0261193 (PMC8789149; doi:10.1371/journal.pone.0261193)
Supplement: S1 File — Contains a block Gibbs sampler, as well as, additional optimal threshold; adjacency heatmaps and graphical network figures for dimensions p = 30 and p = 100. (PDF) [file pone.0261193.s001.pdf]

Supplementary material: Empowering differential networks using Bayesian analysis

Jarod Smith [1¶*], Mohammad Arashi [2,1¶], Andriëtte Bekker [1¶]

[1] Department of Statistics, University of Pretoria, Pretoria, South Africa
[2] Department of Statistics, Faculty of Mathematical Sciences, Ferdowsi University of Mashhad, Mashhad, Iran

* arashi@um.ac.ir (MA)

¶ These authors contributed equally to this work.

## Appendix A: Block Gibbs sampler

The block Gibbs sampler in [1] for the hierarchical representation Eq (7) of Eq (3), allowing for different $\lambda_{ij}$'s in the target distribution Eq (5) is re-presented here for completeness.

To update a single column and row of $\boldsymbol{\Theta}$ per iteration - focusing on the last column and row - let $\boldsymbol{\Upsilon}$ be a $p \times p$ symmetric matrix with a zero main diagonal and $\boldsymbol{\tau}$ in the upper off diagonal entries. Secondly, partition the matrices $\boldsymbol{\Theta}$, $\boldsymbol{S}$ and $\boldsymbol{\Upsilon}$ as follows

$$\boldsymbol{\Theta} = \begin{pmatrix} \boldsymbol{\Theta_{11}} & \boldsymbol{\theta_{12}} \\ \boldsymbol{\theta_{21}} & \theta_{22} \end{pmatrix}, \quad \boldsymbol{S} = \begin{pmatrix} \boldsymbol{S_{11}} & \boldsymbol{s_{12}} \\ \boldsymbol{s_{21}} & s_{22} \end{pmatrix}, \quad \boldsymbol{\Upsilon} = \begin{pmatrix} \boldsymbol{\Upsilon_{11}} & \boldsymbol{\tau_{12}} \\ \boldsymbol{\tau_{21}} & 0 \end{pmatrix}. \tag{1}$$

Notice that the conditional distribution of the last column in Eq (5) can be presented as

$$p(\boldsymbol{\theta_{12}}, \theta_{22} \mid \boldsymbol{\Theta}_{11}, \boldsymbol{\Upsilon}, \boldsymbol{S}, \lambda) \propto (\theta_{22} - \boldsymbol{\theta}_{21}\boldsymbol{\Theta}_{11}^{-1}\boldsymbol{\theta}_{12})^{\frac{n}{2}} \\ \times exp\left[-\frac{1}{2}\{\boldsymbol{\theta_{21}}\boldsymbol{D_{\tau}}^{-1}\boldsymbol{\theta_{12}} + 2\boldsymbol{s}_{21}\boldsymbol{\theta_{12}} + (\lambda + s_{22})\theta_{22}\}\right],$$

here $\boldsymbol{D_{\tau}}$ is the diagonalised matrix of the vector $\boldsymbol{\tau}_{12}$. The following change of variables

$$\boldsymbol{\beta} = \boldsymbol{\theta}_{12}$$
$$\gamma = \theta_{22} - \boldsymbol{\theta}_{21}\boldsymbol{\Theta}_{11}^{-1}\boldsymbol{\theta}_{12}$$

with Jacobian independent of $(\boldsymbol{\beta}, \gamma)$, yields the following conditional distribution

$$p(\boldsymbol{\beta}, \gamma \mid \boldsymbol{\Theta}_{11}, \boldsymbol{\Upsilon}, \boldsymbol{S}, \lambda) \propto (\gamma)^{\frac{n}{2}} exp(-\frac{s_{22} + \lambda}{2}\gamma) \\ \times exp\left[-\frac{1}{2}(\boldsymbol{\beta}^{\top}\{\boldsymbol{\tau}^{-1} + (s_{22+\lambda})\boldsymbol{\Theta}_{11}^{-1}\}\boldsymbol{\beta} + \boldsymbol{2s_{21}\beta})\right].$$

It follows that

$$(\boldsymbol{\beta}, \gamma \mid \boldsymbol{\Theta}_{11}, \boldsymbol{\Upsilon}, \boldsymbol{S}, \lambda) \sim \mathrm{GA}(\frac{n}{2} + 1, \frac{s_{22} + \lambda}{2})\mathrm{N}(-\boldsymbol{C}\boldsymbol{s}_{21}, \boldsymbol{C}),$$

where $\boldsymbol{C} = \{(s_{22}+\lambda)\boldsymbol{\Theta}_{11} + \boldsymbol{D}_{\boldsymbol{\tau}}^{-1}\}^{-1}$. See [1] for a discussion on how the positive definite constraint on $\boldsymbol{\Theta}$ is adhered to in the formulation above.
Moreover, $\boldsymbol{\tau}$ can be updated by observing that the conditional posterior distribution of the $1/\tau_{ij}$'s in Eq (5) are independently inverse Gaussian (INV − GAU) with $\lambda_{ij}^{'} = \lambda_{ij}^{2}$ and $\mu^{'} = \sqrt{(\lambda_{ij}^{2}/\theta_{ij}^{2})}$

$$p(x) = (\frac{\lambda_{ij}^{'}}{2x\pi^{3}})^{\frac{1}{2}} exp\bigg\{\frac{-\lambda_{ij}^{'}(x-\mu^{'})^{2}}{2(\mu^{'})^{2}x}\bigg\}, \quad x > 0.$$

The block Gibbs sampler algorithm is summarised by

**Algorithm 1** Block Gibbs sampler

1: Set the hyperparameters $(s, r)$ for the gamma distribution in Eq (7) and initialise $\{\lambda_{ii}\}_{i=1}^{p}$ and $\boldsymbol{\tau}$.
2: **for** $i = 1, 2, \ldots, p$ **do**
3: Partition $\boldsymbol{\Theta}$, $\boldsymbol{S}$ and $\boldsymbol{\Upsilon}$ as in Eq (1).
4: Sample $\gamma \sim \mathrm{GA}(n/2+1, (s_{22}+\lambda)/2)$ and $\boldsymbol{\beta} \sim \mathrm{N}(-\boldsymbol{C}\boldsymbol{s}_{21}, \boldsymbol{C})$.
5: Update $\boldsymbol{\theta}_{12} = \boldsymbol{\beta}, \boldsymbol{\theta}_{21} = \boldsymbol{\beta}^{\top}, \theta_{22} = \gamma + \boldsymbol{\beta}^{\top}\boldsymbol{\Theta}_{11}^{-1}\boldsymbol{\beta}$
6: **end for**
7: **for** $i \neq j$ **do**
8: Sample $\lambda_{ij} \sim \mathrm{GA}(1+r, |\theta_{ij}| + s)$.
9: Sample $\delta_{ij} \sim \mathrm{INV-GAU}(\mu^{'}, \lambda^{'})$ and update $\tau_{ij} = 1/\delta_{ij}$
10: **end for**

For the choice of the hyperparameters $(r, s)$, [1] suggest $r = 10^{-2}$ and $s = 10^{-6}$ for excellent performance in the adaptability of $\lambda_{ij}$ to each $\theta_{ij}$ and $\lambda_{ii} = 1$.

# Appendix B: Additional synthetic data analysis figures

Figs 1 and 2 provide additional insight into the comparative performance of the of the B-net estimator with regards to the estimated scale-free, band and cluster network structures. The best suited sparsity threshold for $p = 30$ for said structures is presented in Fig 5. Similarly, Figs 3, 4 and 6 depict the aforementioned insights for $p = 100$.

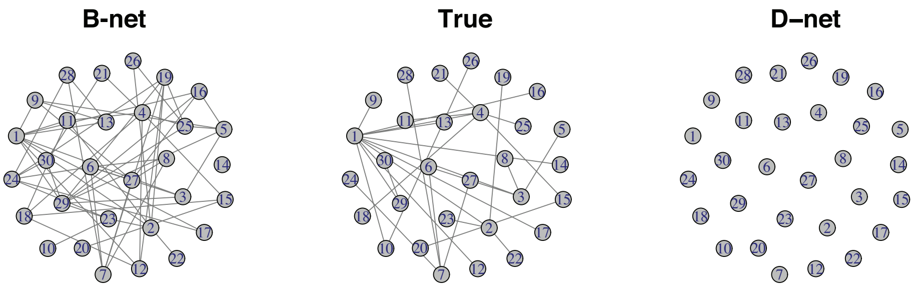


**(a)** Model 5: scale-free.

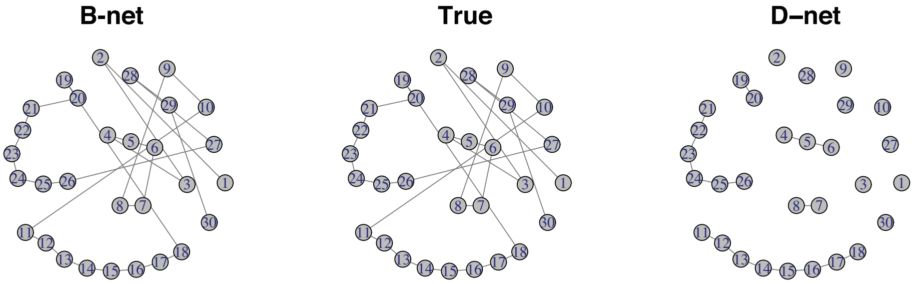


**(b)** Model 6: band.

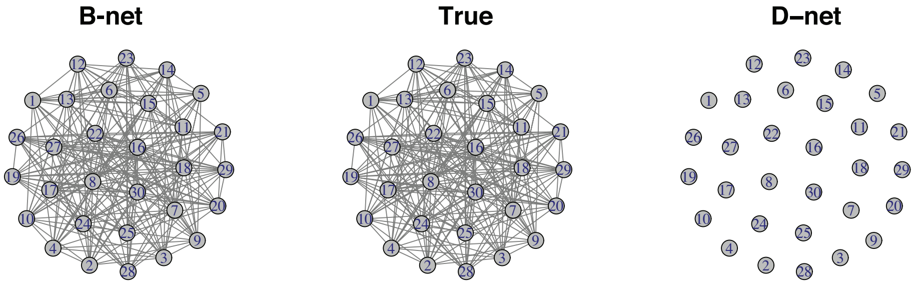


**(c)** Model 7: cluster.

**Fig 1. Comparison of the true DN, B-net and D-net graphical structure estimates for the scale-free, band and cluster structure for $p = 30$.**

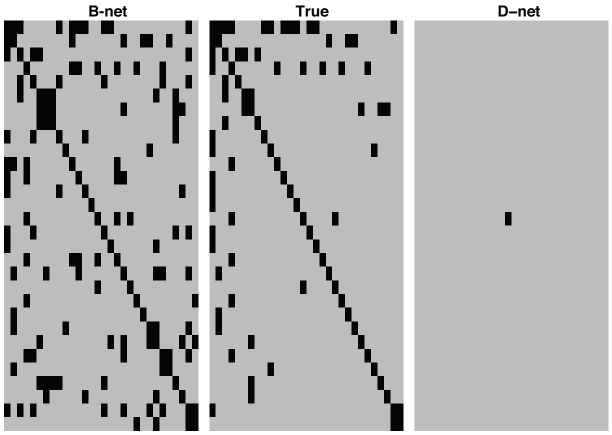


**(a)** Model 5: scale-free.

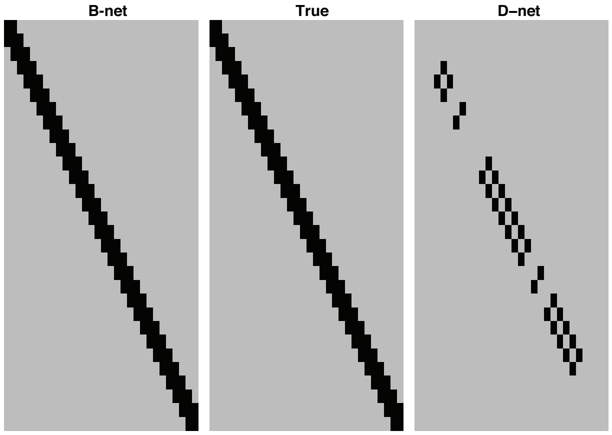


**(b)** Model 6: band.

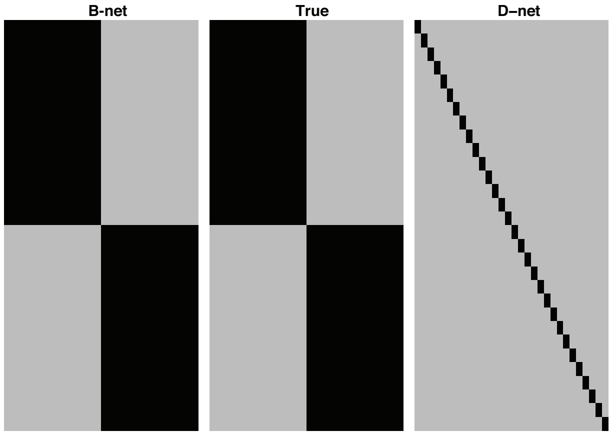


**(c)** Model 7: cluster.

**Fig 2. Comparison of the true DN, B-net and D-net adjacency matrix estimates for the scale-free, band and cluster structure for $p = 30$.**

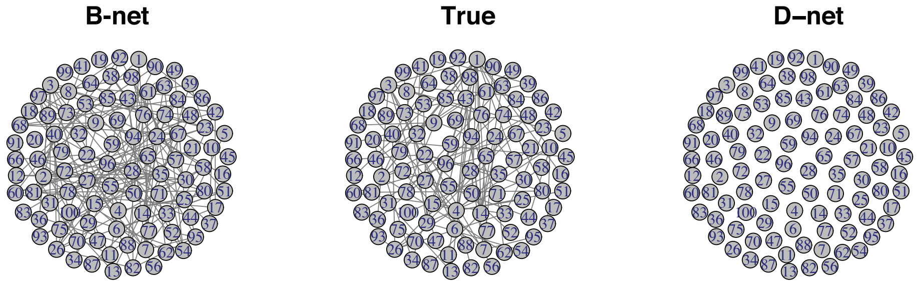


**(a)** Model 5: scale-free.

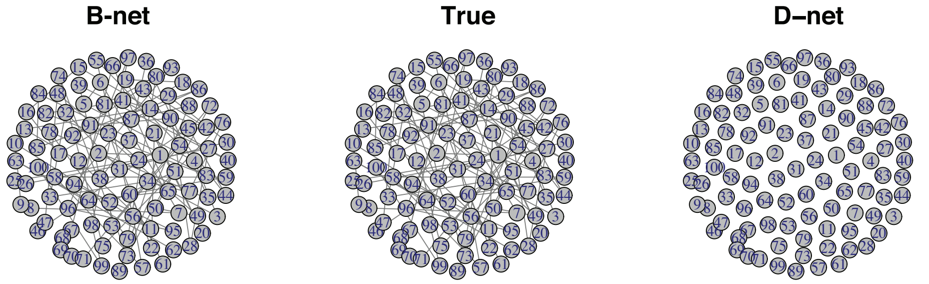


**(b)** Model 6: band.

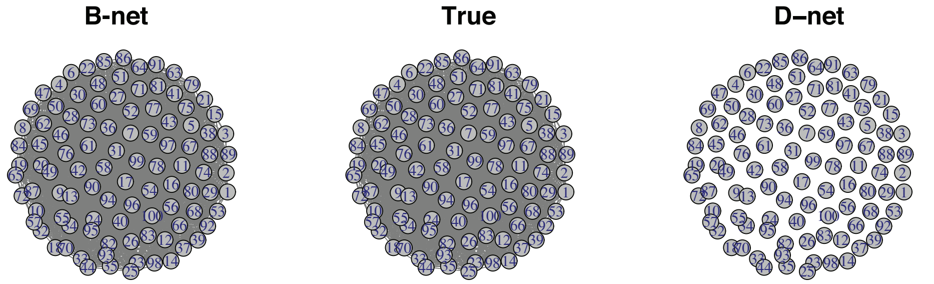


**(c)** Model 7: cluster.

**Fig 3. Comparison of the true DN, B-net and D-net graphical structure estimates for the scale-free, band and cluster structure for $p = 100$.**

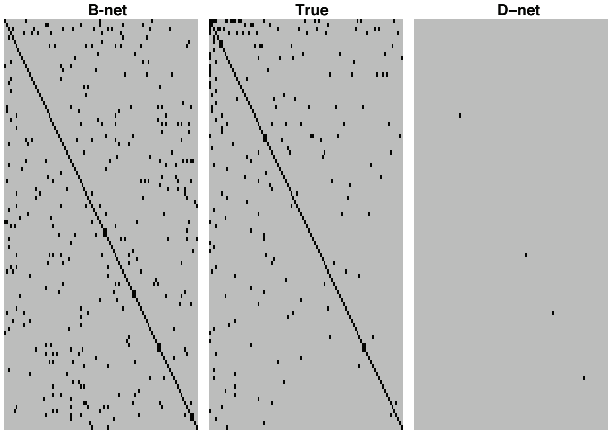


**(a)** Model 5: scale-free.

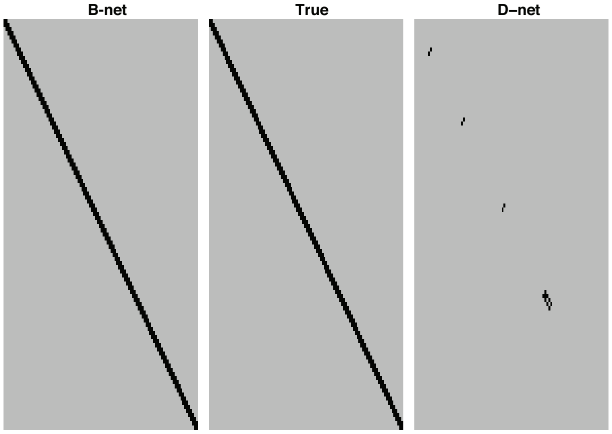


**(b)** Model 6: band.

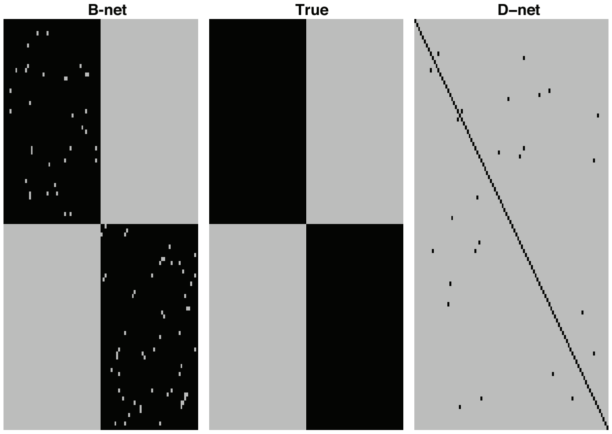


**(c)** Model 7: cluster.

**Fig 4. Comparison of the true DN, B-net and D-net adjacency matrix estimates for the scale-free, band and cluster structure for $p = 100$.**

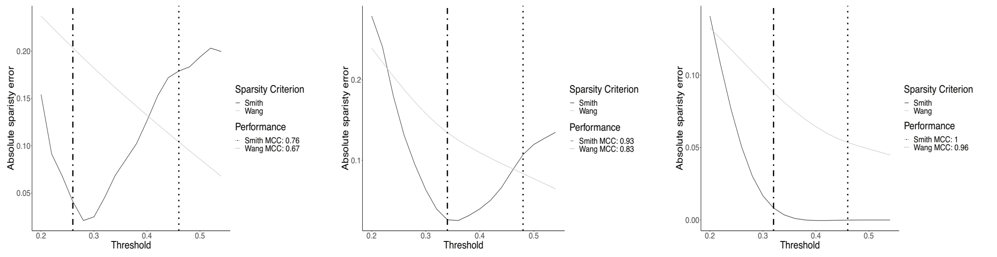


**(a)** Model 5: scale-free. **(b)** Model 6: band. **(c)** Model 7: cluster.

**Fig 5. Optimal Bayesian sparsity threshold selection for $p = 30$.** The median of the absolute sparsity error and best performing MCC for the scale-free, band and cluster structure under varying thresholds for each Bayesian sparsity criterion in Eq (9) (dotted) and Eq (8) (dot-dash) for dimension $p = 30$. The best performing threshold is indicated by a vertical line with the accompanying MCC value displayed in the legend.

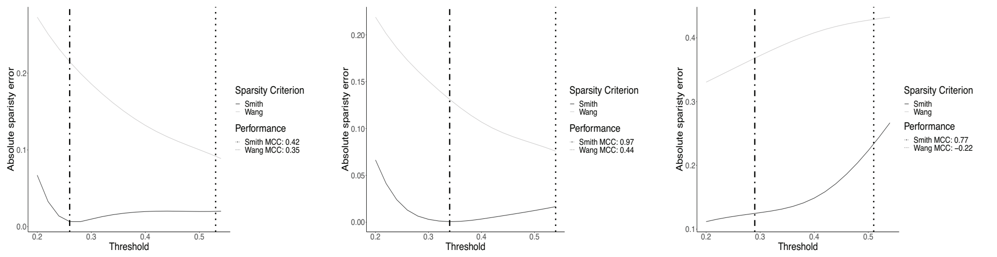


**(a)** Model 5: scale-free. **(b)** Model 6: band. **(c)** Model 7: cluster.

**Fig 6. Optimal Bayesian sparsity threshold selection for $p = 100$.** The median of the absolute sparsity error and best performing MCC for the scale-free, band and cluster structure under varying thresholds for each Bayesian sparsity criterion in Eq (9) (dotted) and Eq (8) (dot-dash) for dimension $p = 100$. The best performing threshold is indicated by a vertical line with the accompanying MCC value displayed in the legend.

# References

1. Wang H. Bayesian graphical lasso models and efficient posterior computation. Bayesian Analysis. 2012;7(4):867–886.
